# Does picture background matter? Peopleʼs evaluation of pigs in different farm settings

**DOI:** 10.1371/journal.pone.0211256

**Published:** 2019-02-12

**Authors:** Gesa Busch, Sarah Gauly, Marie von Meyer-Höfer, Achim Spiller

**Affiliations:** 1 Agricultural and Food Economics, Faculty of Science and Technology, Free University of Bozen-Bolzano, Bozen-Bolzano, Italy; 2 Department of Agricultural Economics and Rural Development, Faculty of Agricultural Sciences, University of Goettingen, Goettingen, Germany; Memorial University of Newfoundland, CANADA

## Abstract

Pictures of farm animals and their husbandry systems are frequently presented in the media and are mostly connected to discussions surrounding farm animal welfare. How such pictures are perceived by the broader public is not fully understood thus far. It is presumable that the animalsʼ expressions and body languages as well as their depicted environment or husbandry systems affect public perception. Therefore, the aim of this study is to test how the evaluation of a picture showing a farmed pig is influenced by portrayed attributes, as well as participants’ perceptions of pigs’ abilities in general, and if connection to agriculture has an influence. In an online survey, 1,019 German residents were shown four modified pictures of a pig in a pen. The pictures varied with regards to facial expression and body language of the pig (ʽhappyʼ versus ʽunhappyʼ pig) and the barn setting (straw versus slatted floor pen). Respondents were asked to evaluate both the pen and the welfare of the pig. Two Linear Mixed Models were calculated to analyze effects on pig and pen evaluation. For the pictures, the pen had the largest influence on both pig and pen evaluation, followed by the pigʼs appearance and participants’ beliefs in pigs’ mental and emotional abilities, as well as their connection to agriculture. The welfare of both the ʽhappyʼ and the ʽunhappyʼ pig was assessed to be higher in the straw setting compared to the slatted floor setting in our study, and even the ʽunhappy pigʼ on straw was perceived more positively than the ʽhappy pigʼ on slatted floor. The straw pen was evaluated as being better than the slatted floor pen on the pictures we presented but the pens also differed in level of dirt on the walls (more dirt in the slatted floor pen), which might have influenced the results. Nevertheless, the results suggest that enduring aspects of pictures such as the husbandry system influence perceptions more than a momentary body expression of the pig, at least in the settings tested herein.

## Introduction

The environment in which an animal is presented has a clear effect on the characteristics that people ascribe to the animal [1, 2, 3]. Rhoads and Goldsworthy [2] analyzed the perception of pictures of zoo animals in different settings (in the wild vs. in a zoo) and found that the setting has a clear effect on peoples’ perceptions of the animal [2]. Finlay et al. [3] confirmed this finding, indicating that the same animal was associated with different attributes if set in a zoo environment compared to a natural habitat, with more positive attributions being ascribed in the wilderness setting. To our best knowledge, there are no comparable studies which focus on peoples’ perceptions of pictures showing farm animals in different settings, such as various husbandry systems. Nevertheless, for farm animals, especially pigs, it is known that the husbandry system can influence the qualitative assessment of a pig’s behavior; e.g., pigs shown in an outdoor environment were rated as being more playful/active and less bored/lethargic by veterinary students [4].

The impact of the background setting can also be influential when people view pictures of farm animals in the media, as presently, media exposure is the primary source of communication about livestock farming for the general public [5, 6, 7]. Thus, investigating how these pictures are perceived and what influences picture evaluation may provide interesting insights into the recent debate about farm animal welfare. In this context, husbandry systems for pigs are highly discussed among the general public [5, 7, 8]. When people have the opportunity to evaluate pig husbandry systems, housing and floor type have been shown to influence their evaluation [9, 10, 11] and therefore contribute to the public image of a system. Litter bedding, such as straw, is usually perceived to be better for pigs than slatted floors and a positive impact on the animals’ well-being is expected [7, 9, 10, 12]. Nevertheless, slatted floor systems are currently the most common housing system for pigs in European countries. In Germany, for instance, which is the largest pig production country in the EU [13], over 90% of all pigs are kept in barns with either fully or partially slatted floors, whereas barn systems with bedding material or outside access are comparably scarce [14].

When people view pictures of animals, not only the background matters. Rather, a picture is processed by the viewer as an interaction between the object and its background [15]. With respect to pictures of animals, it is obvious that not only the background, but the animal itself with its facial expression and body language has the potential to impact perception and evaluation. The manner of expressing emotions in the face is highly species-specific, and humans can easily interpret other human facial expressions [16]. Besides paying attention to facial behavior of other conspecifics, humans are also sensitive to the emotional expression of non-human animals [17]. Thus, studies provide evidence that humans can correctly interpret facial expressions of dogs and their ratings follow similar patterns as the ratings for human facial expressions [16, 17, 18]. This indicates that the detection of affective states in animals is also, at least for some animals, possible from evaluating only their face and body language. Studies analyzing facial expressions in animals are becoming increasingly prevalent, with one goal being to detect pain using grimace scales (e.g., for horses: [19]; for mice: [20]; for rats: [21]). Moreover, there are approaches to interpret animal emotions using the animalsʼ expressions and gestures to draw conclusions on behavior and emotions. For example, in qualitative behavior assessment approaches, the animals’ body language is used to analyze animal behavior–an approach which is based on human perceptions of animals (e.g., for dairy cattle: [22]; for pigs: [23]). However, even if it has been shown that humans are able to interpret facial expressions of some species correctly, no studies so far have investigated how people from the broader public interpret farm animals’ facial expressions and body language and in which way their evaluation influences their opinion on the welfare of the animal. It is likely that it is more difficult to assess emotional states of farm animals than those of domesticated dogs, due to less familiarity with farm animals. Although there is evidence that familiarity with dogs is not necessary to correctly detect their basic facial expressions [16, 17], Schirmer et al. [16] conclude that experience with dogs facilitates the interpretation of their emotional state in more complex situations, including the assessment of body gestures. Considering the fact that only few approaches exist to reliably determine emotion-related facial expressions in farm animals by experts [24], it appears to be even more difficult for lay people. However, even if peoples’ conclusions fail to recognize the real affective states of the farm animals, perceived emotions shape their evaluation of the animalsʼ well-being [18]. Thus, it is crucial to know which emotional states people perceive in order to understand their concerns towards animal welfare issues [18]. Further, studies have shown that general attitudes towards animals’ mental and emotional capacities influence peoples’ attitudes about how to use and treat such animals [25, 26] and this might also contribute to the perception and evaluation of pictures showing farm animals.

Therefore, the present study investigates whether the perception of a pig in a given husbandry system and of a pen with a pig in it is influenced by the following: 1) the barn setting (straw floor pen vs. slatted floor pen) and 2) the body language and facial expression of the pig (ʽhappyʼ vs. ʽunhappyʼ expression). We further analyze if attitudes towards pigs’ mental abilities influence perceptions of the pigs and the pens.

The order in which questions are presented to respondents in surveys has been shown to influence particpants’ responses [27]. If people answer attitude questions, so-called judgemental carryover effects can occur [28]. This means that standards for comparison that have been used by participants to answer a question are also applied as comparison in answering questions that follow in the questionnaire. It is therefore possible that the order in which different pictures are presented influences the evaluation of the pictures. For example, a picture showing a more negatively perceived husbandry system such as the slatted floor might lead to a more positive evaluation of a straw pen picture because it is used as frame of reference. Thus, this experiment further tests if the order in which the pictures are presented has an influence on their evaluation. People who are more connected to agriculture, e.g. those who grew up on a farm, often show a more positive attitude towards current farming methods compared to people with lower involvement in agriculture [29; 30]. Therefore, we also evaluated the influence of participants’ connections to agriculture on picture perception.

## Materials and methods

### Survey design

**Fig 1:**
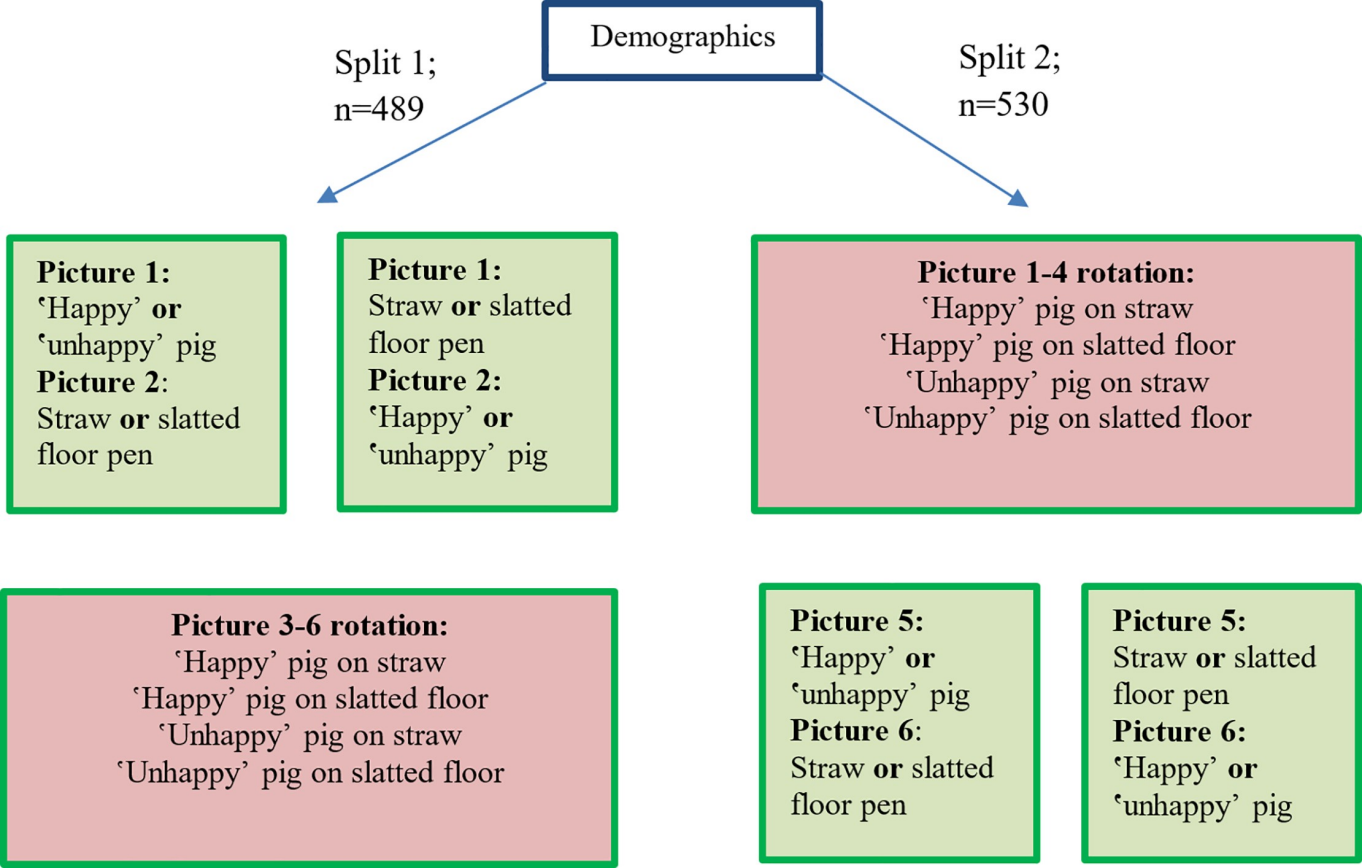
Survey design and sample splits. The order of pictures 1 and 2 (or 5 and 6) as well as pictures 3–6 (or 1–4) was randomized for each sample split.

To investigate the influence of the perceived body language of pigs and the barn setting on picture perception, different pictures were presented to members of the non-farming public in an online-survey. Both the pig and the barn had two variations: the expression of the pig could be either ʽhappyʼ or ʽunhappyʼ and the barn could be either a pen with straw bedding or a pen with slatted floor. Four pictures showing combinations of both attributes, the ʽhappyʼ- or ʽunhappyʼ-looking pig in a pen with slatted floor or straw bedding, respectively, were used in the survey. All participants saw each of the four possible combinations of pig and pen. After viewing each picture, participants were asked to evaluate the scene on semantic differential scales. The order of the pictures was randomized for each participant. In order to establish a baseline for the evaluation, participants were also asked to evaluate one of the pig pictures (without the barn setting) and one of the pen pictures (without the pig in it) separately; the order of these were also randomly selected, i.e., the sample was randomly split into two sub-samples of which one group of participants first saw the pictures of the pig and the pen separately (split 1, n = 489), while the other group evaluated the four combined pictures including pen and pig before evaluating the pig and pen picture separately (split 2, n = 530). Fig 1 illustrates the survey design.

The questionnaire consisted of two parts (see Fig 1). In the first part, respondents were asked about their gender, age, income and level of education, as well as about their connection to agriculture and how often they had previously visited pig barns. Further, people were questioned about their belief in pigs’ mind using six statements about mental and emotional abilities of animals following Hills [31]. The second part of the questionnaire is the core of the study and consisted of the evaluation of the six pictures described above.

For each picture, participants were asked to evaluate both the pig and the pen on a semantic differential scale showing opposing word pairs. For each of the word pairs, respondents could indicate their answer on a five-point scale between two opposing poles (e.g., ʽsatisfiedʼ and ‘unsatisfied’). For data analysis, answers were coded from 1 (most positive) to 3 (neutral) to 5 (most negative). Based on the literature about aspects contributing to the evaluation of animal welfare by the non-farming public, six word pairs were selected for the evaluation of each, the pig and the pen, respectively (see Table 1). These word pairs are intended to provide a comprehensive overview of the perceived welfare of the animal and the evaluation of the pen from the respondents’ perspective.

**Table 1:** Word pairs used for the evaluation of the pigs and pens (on a five-point semantic differential) and reasons for choosing the word pairs (derived from literature).

**Word pairs for pig evaluation**	**Reason for choosing the word pair**
Satisfied—unsatisfied	The level of satisfaction is used by citizens to evaluate animals’ well-being [7]
Happy—unhappy	People link animal well-being to happiness [7]
Relaxed—stressed	Affective states such as stress are mentioned by the public when evaluating housing systems for pigs [8]
Active—passive	Animals’ degree of activity influences people’s feelings towards animals [32]
Healthy—sick	Health is considered to be one of the most important factors for the well-being of an animal from a consumers’ perspective [33]
Brave—anxious	Consumers consider an animal’s experience of little or no fear to be an important factor when judging animal husbandry systems [33]
**Word pairs for pen evaluation**	
Species-appropriate—not species-appropriate	People view the opportunity for animals to realize species-specific behavior as being essential for a good animal life [5]
Natural—unnatural	Naturalness of husbandry systems plays an important role for the public when considering the well-being of farm animals [33]
Comfortable—uncomfortable	Comfortable living conditions are associated with animal-friendly husbandry systems by consumers [33]
Future-proof—not future-proof	Public acceptance of future livestock farming is important for its sustainability [34]
Profit-oriented—not profit-oriented	Public concern is that farmers may focus more on efficiency and profit rather than on the welfare of animals [35]
Clean—dirty	Cleanliness of the environment is considered to be important when investigating consumer attitudes towards the development of animal-friendly husbandry systems [33]

### Pictures

**Fig 2:**
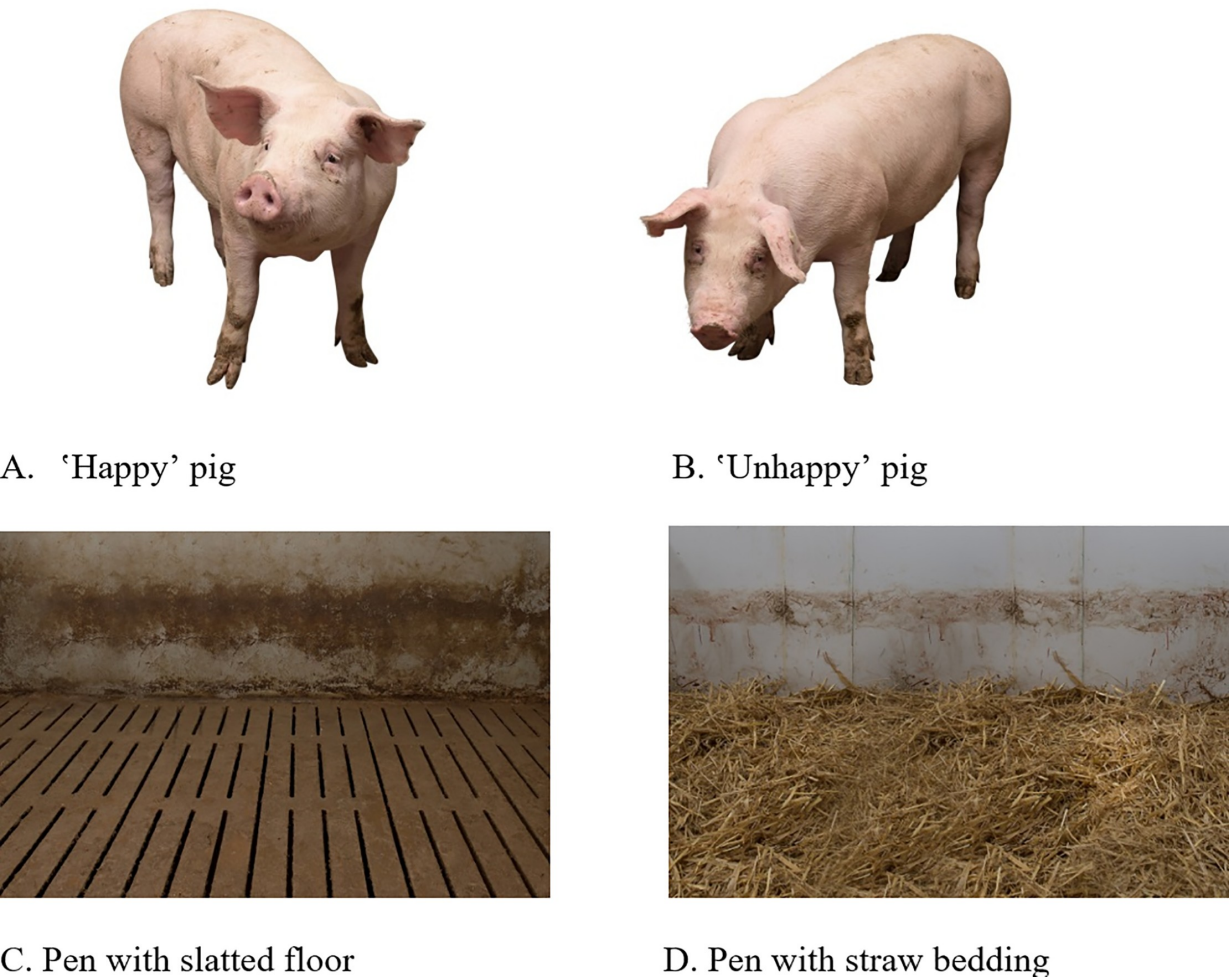
Pictures of pigs and pens presented as stimuli to survey participants. Source: Landpixel (Swen Pförtner).

The pictures used in the study were taken by a photographer specializing in agricultural photography. For the pictures of the pig, snapshots from different pigs were taken in a conventional pig barn. Two pictures were selected based on a pretest with 41 participants that were conveniently sampled via Facebook, where the only prerequisite for taking part was having no connection to agriculture. In the pretest, participants evaluated seven snapshots on a seven-point semantic differential using ʽhappyʼ and ʽunhappyʼ as poles for the evaluation of the pigs. The two pictures rated as the ʽhappiestʼ-looking and the ʽunhappiestʼ-looking pig, were chosen for the study presented herein (see Fig 2, pictures A and B). In the following, the terms ‘happy’ and ‘unhappy’ are used for describing both pigs, but it is important to note that this wording reflects the evaluation of lay people in the pretest and does not necessarily reflect the affective state of the animal when the pictures were taken. Additionally, two pictures of empty pens were taken; one with a slatted floor and one with straw bedding (see Fig 2, pictures C and D). Although we tried to be keep the two pictures as similar as possible to each other (aside from the flooring conditions), the level of dirt on the walls differs between the two pictures. The strip of dirt appears to be larger and darker in the slatted floor pen compared to the straw pen. This difference became apparent only after data were collected and therefore couldn’t be changed for the study presented herein; however, it must be kept in mind when interpreting the results.

**Fig 3:**
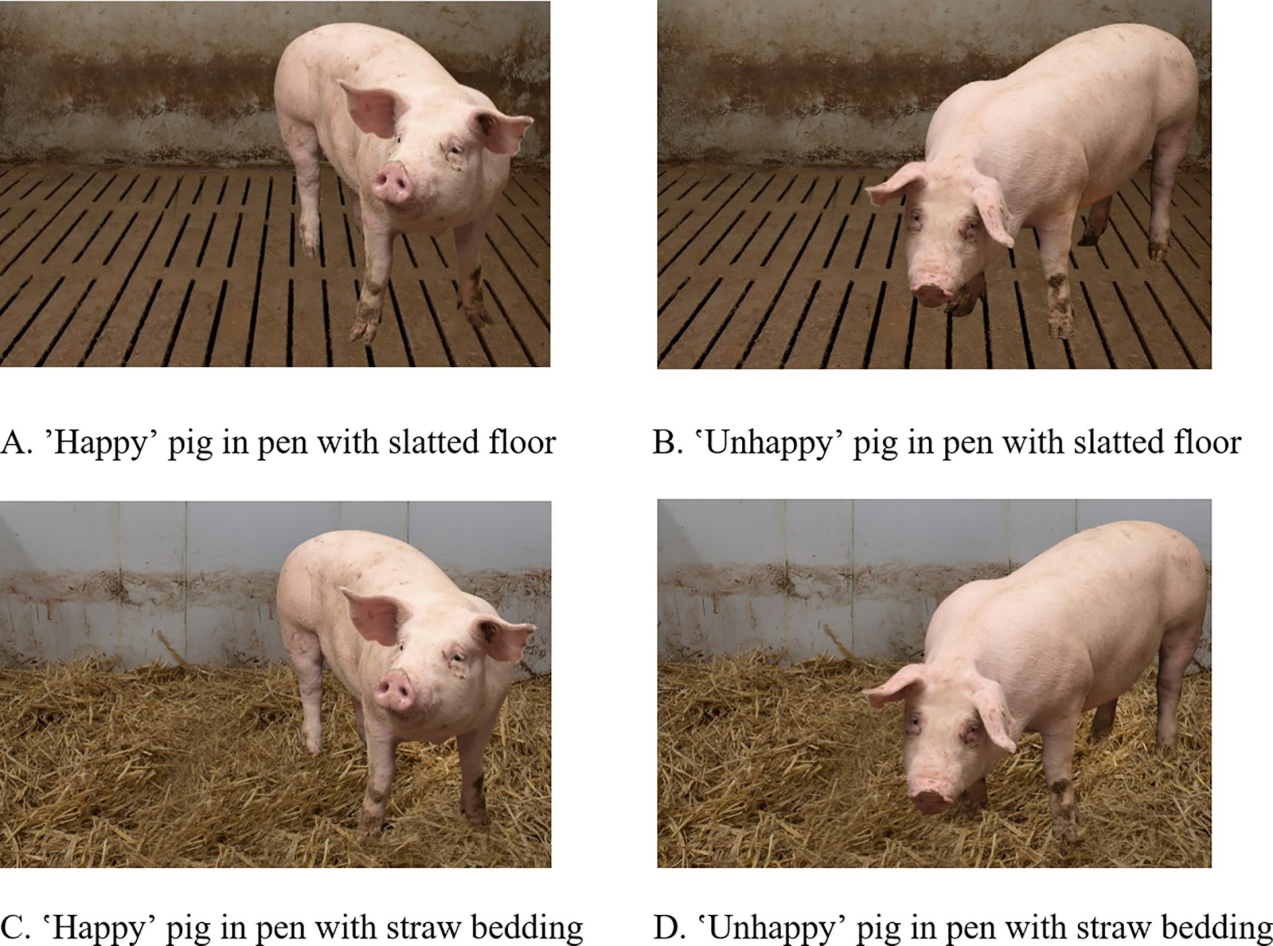
Combined pictures of pigs and pen settings presented as stimuli to survey participants. Source: Landpixel (Swen Pförtner).

Using Adobe Photoshop, the pictures of the pigs and the pens were combined into four pictures showing the ʽhappyʼ and ʽunhappyʼ pig in both barn settings (see Fig 3).

In the end, eight pictures were used for the survey (see Figs 2 and 3): two pictures showing only the pigs (ʽhappyʼ and ʽunhappyʼ), two pictures showing only the pens (slatted floor and straw bedding) and four combined pictures showing the ʽhappyʼpig in the straw pen, the ‘happy’ pig in the slatted floor pen, the ʽunhappyʼ pig in the straw pen and the ‘unhappy’ pig in the slatted floor pen. Each participant viewed six pictures in total consisting of the four combined pictures (Fig 3), as well as one randomly selected picture of the pig and one of the pen (Fig 2).

### Participant recruitment and data collection

The study is based on an online survey that was conducted in June and July 2016 with German residents. Quotas were set for gender, age, income and education and participants were recruited using an online access panel provider (Norstat AS). In total, 1,071 respondents participated in the survey. To ensure that participants read all questions thoroughly, two quality check questions were incorporated over the course of the survey. Respondents simply had to select the answer that was requested within the quality check question (e.g., “Please tick the very leftmost box” on the semantic differential). Participants who failed to correctly answer these two questions were excluded from the dataset. Those who did not complete the entire survey were also excluded. In an additional quality check, ‘straightliners’ [36] and participants that answered the survey in less than half of the average response time were removed. In the end, the data from 1,019 participants remained for analysis.

### Ethics statement

This study was conducted in full accordance with the Ethical Principles of the German Psychological Society (DGP) and the Association of German Professional Psychologists (BDP). At the time that the data were acquired in June and July of 2016, it was not customary at Goettingen University, nor at most other German universities, to seek ethical approval for studies related to attitudes and picture perception, such as the current study. The survey was designed by the authors in a way that would avoid distressing or negatively impacting participants. The confidentiality of collected data was assured by the online access panel provider used for participant recruitment in accordance with the general data protection regulation and the legal guidelines of the European Society of Opinion and Marketing Research (ESOMAR), the Federation of German Market and Social Researchers (BVM), as well as the Association of German Market and Social Research Institutes e. V. (ADM). Furthermore, data acquisition, data processing and utilization of personal data were conducted in full accordance with the strict guidelines of the German Bundesdatenschutzgesetz (BDSG).

All participants confirmed voluntary participation via email and had the right to withdraw from the study at any time simply by closing their internet browser. The study exclusively utilizes anonymous questionnaires. No data can be identified and/or linked to individual participants and all participants were informed that data would be anonymously analyzed.

### Data analysis

The data was analyzed using IBM SPSS Statistics 25. Differences in the sociodemographic data between the two splits (one group who saw the pictures of the pig and the pen separately first and one group who saw the composition pictures showing the pig and the pen together first) were analyzed using cross tabulations and Chi-square tests. Using ANOVA, the mean evaluations of the pigs and pens on all eight pictures used in the study are described and compared. We conducted a factor analysis with varimax rotation to condense data on particpants’ belief in pig mind. Two factors could be extracted, each containing three of the six statements (total variance explained: 55.81%, Kaiser-Meyer-Olkin Measure of Sampling Adequacy = 0.727, Bartletts’ test of sphericity = 0.000, Cronbachʼs Alpha factor 1: 0.61, Cronbachʼs Alpha factor 2: 0.58). Further information is displayed in the results section. We analyzed differences between the splits regarding participants’ belief in pig mind using independent samples t-test with an alpha level of 0.05. Means and standard deviations are given in the results section.

Further, two linear mixed models were used to analyze the effects of the pig, the pen, the split, the order of combined pictures and participants’ connection to agriculture as well as their belief in pigs’ mind on evaluation of pigs and pens on the four combined pictures. In the first model, the dependent variable is an unweighted index for pig evaluation that is based on the arithmetic means of the six word-pairs for pig evaluation described above. In the second model, the dependent variable is an unweighted index for pen evaluation that is based on the arithmetic means of five word-pairs for pen evaluation. Both indices range from 1 to 5 with 1 = positive evaluation of the pig/pen and 5 = negative evaluation of the pig/pen. As a prerequisite for summing up the evaluation scores of the word pairs into the indices, we conducted two factor analyses with varimax rotation: one for the evaluation of the pig and one for the evaluation of the pen according to the six word pairs (see Table 1) and found one factor for each based on Eigenvalues > 1 (factor pig: total variance explained: 76.94%, Kaiser-Meyer-Olkin Measure of Sampling Adequacy = 0.924, Bartletts’ test of sphericity = 0.000, Cronbachʼs Alpha: 0.94; factor pen: total variance explained: 79.69%, Kaiser-Meyer-Olkin Measure of Sampling Adequacy = 0.887, Bartletts’ test of sphericity = 0.000, Cronbachʼs Alpha: 0.94). All word pairs show high factor loadings on the respective factors (above 0.8 for the evaluation of the pig and above 0.7 for the evaluation of the pen), except for the word pair ‘profit-oriented–not profit-oriented’ which did not load on the factor “evaluation pen” and was therefore excluded from further analyses. Further details on the results of the factor analyses can be found in S1 File.

The indices representing overall evaluation of the pig and the pen were each introduced as dependent variables in the two linear mixed models. Picture composition (ʽhappyʼ or ʽunhappyʼ pig, straw or slatted floor), the order of the pictures as presented to participants in the study (separate or combined pictures were seen first; (split)), the first combined picture that a participant viewed (first picture), participants connection two agriculture (if they grew up on a farm (grew up on farm) and if they have no connection to agriculture (no connection ag)), as well as the two factors representing participants’ belief in pig mind, were introduced as fixed effects in the models. We further included the interaction of pig x pen. The participant was introduced as a random effect, resulting in the following statistical model:

$$Y_{iklmnopqr}=\mu +{Pig}_{i}+{Pen}_{j}+{\left(Pig\;x\;Pen\right)}_{ij}+{Split}_{k}+{First\;Picture}_{l}+{Grew\;up\;on\;farm}_{m}+{No\;connection\;ag}_{n}+{Belief\;in\;Pig\;Mind\;I}_{o}+{Belief\;in\;Pig\;Mind\;II}_{p}+{Participant}_{q}+e_{ijklmnopqr}$$

where **Y**_**ijklmnopqr**_ is the r^th^ evaluation of the Pig i or Pen j in Split k with the First Picture l, Grew up on farm_m_, No connection to ag_n_ and the Belief in Pig Mind I_o_ and Belief in Pig Mind II_p_ of participant q; μ is the intercept. Pig_i,_ Pen_j,_ Split_k_, First Picture_l,_ Grew up on farm_m_, No connection ag_n_ are the fixed effects for pig expression i (i = happy, unhappy looking pig), pen design j (j = straw pen, slatted floor pen), sample split k (k = combined pictures first, solo pictures first), the first combined picture that has been seen l (l = straw/unhappy, straw/happy, slatted floor/happy, slatted floor/unhappy), if participants’ grew up on a farm m (m = grew up on a farm, did not grow up on a farm), participants’ self stated connection to agriculture n (n = no connection to agriculture, somehow a connection to agriculture) and the interaction between pig i and pen j. Belief in Pig Mind I o and Belief in Pig Mind II p are the covariate fixed effects. Participant q is the random effect of participant (1–1019) and e_ijklmnopqr_ is the residual error.

The effects of pig, pen, growing up on a farm, connection to agriculture and split on pig and pen evaluation were analyzed using Least Squares (LS) Means comparison with F-Test and with an alpha level set at 0.05.

### Sample description

According to quotas that were set for gender, age, income and education, both sample splits are very close to the actual distribution of the German population (see Table 2). We tested if the two splits differ in demographics: To account for participants’ (potential) connections to agriculture it can be noted that in split 1 and 2, 8.0% and 12.5% grew up on a farm (p≤0.05), 0.8% and 1.7% stated that they work in agriculture (p>0.05), 8.4% and 7.7% that a family member works in agriculture (p>0.05), and 0.8% and 0.9% that they completed an agricultural career (p>0.05), respectively. In total, 59.1% in split 1 and 52.3% in split 2 affirmed that they have no connection to agriculture (p≤0.05). With regard to the frequency of having been in a pig barn, 19.2% of participants in split 1 and 17.5% of participants in split 2 indicated that they have never been in a pig stable before (p>0.05) and 68.5% (split 1) and 66.3% (split 2), respectively, stated that they have only been in such a stable once or several times (p>0.05). Regarding meat consumption, 4.1% of participants in split 1 are vegetarians compared to 5.7% in split 2 (p>0.05). In contrast, 68.9% of respondents in split 1 and 67.7% of respondents in split 2 eat meat on a daily basis or several times per week (p>0.05). To conclude, it can be determined that no major differences between the two splits could be detected apart from participants’ connection to agriculture in which Split 1 and 2 differ in the mentioned aspects. For further data analysis, we will consider the whole sample (n = 1,019), unless stated otherwise.

**Table 2:** Distribution of demographics in the two sample splits (evaluation of single pig and pen pictures first (n = 489) and evaluation of combined pictures first (n = 530)) in comparison to census data from Germany.

Quota	Specification	Split 1 (Single pictures first)	Split 2 (Combined pictures first)	German population
Gender^1^	Male	49.7%	50.2%	49%
	Female	50.3%	49.8%	51%
Age^1^	16–29	18.0%	19.1%	18.7%
	30–49	32.7%	31.5%	31.3%
	50+	49.3%	49.4%	50.0%
Net household income per month^1^	< 1,300€	23.1%	24.5%	23.7%
	1,300 to just under 2,600€	39.9%	38.3%	38.6%
	2,600 to 4,500€	27.6%	25.7%	26.0%
	> 4,500€	9.4%	11.5%	11.8%
Education^1^	No graduation (yet)	1.4%	0.6%	7.1%
	Certificate of Secondary Education	35.4%	36.2%	32.9%
	General Certificate of Secondary Education	31.5%	34.7%	29.4%
	General qualification for university entrance	14.1%	12.5%	14.3%
	University degree	17.6%	16.0%	16.3%

Source: Own calculations; census data from Germany [37, 38]

^1^no significant differences between the two splits.

## Results

### Participants’ belief in pigs’ minds

The degree of participants’ belief in mental and emotional abilities of pigs was surveyed using six statements (Table 3). For 86% of respondents pigs are for sure or probably capable of experiencing emotions and for the majority (57.8%) pigs are rather conscious of what is happening to them. The agreement decreases when asked about pigs’ abilities to solve problems (46%), the ability to see cause and effect of an action (26.8%) and if they are just responding to instinctive urges (41.7%). The highest uncertainty can be observed regarding the question of whether pigs experience emotions less intensly than humans or not. Due to the differences in the two splits with regard to their connection to agriculture, we tested if participants in split 1 and 2 differ in the level of belief in pigs’ mind using ANOVA. With regard to Factor 1 participants in split 1 and 2 differ from each other with split 1 having a stronger belief in pigs’ mind (μ_split1_ = -0.03 (σ = 0.97), μ_split2_ = 0.03 (σ = 1.03); p≤0.05). No differences for Factor 2 between the splits are observed (μ_split1_ = -0.01 (σ = 0.96), μ_split2_ = 0.01 (σ = 1.03); p>0.05).

**Table 3:** Responses to the six statements measuring belief in pigs’ mind in % of participants (N = 1,019) and results of factor analysis for the six statements.

	Yes, for sure.	Probably yes.	I am not sure.	Probably no.	No, for sure not.	Factor loading
Factor I						
Pigs are able to think to some extend to solve problems and make decisions about what to do.	10.9%	35.1%	34.6%	14.0%	5.3%	0.76
Pigs are capable of experiencing a range of emotions (e.g. pain, suffering, contentment, maternal affection, aggression…)	49.6%	36.4%	11.4%	1.8%	0.9%	0.73
Pigs are conscious and aware of what is happening to them.	20.6%	37.2%	28.1%	11.4%	2.7%	0.71
Factor II						
Pigs have limited abilities to see cause and effect of an action.	5.0%	21.8%	39.4%	23.7%	10.2	0.82
Pigs experience emotions less intensly than humans.	3.1%	12.5%	42.0%	27.3%	15.1%	0.69
Pigs are more automatically responding to instinctive urges without awareness of what they are doing.	7.6%	34.1%	33.9%	20.9%	3.6%	0.64

Factor analysis: total variance explained: 55.81%, Kaiser-Meyer-Olkin Measure of Sampling Adequacy = 0.727, Bartletts’ test of sphericity = 0.000, Cronbachʼs Alpha factor 1: 0.61, Cronbachʼs Alpha factor 2: 0.58.

Source: Own calculations.

### Evaluations of pigs and pens

Table 4 shows how the pigs, either presented separately (Fig 2) or with a pen as picture background (Fig 3) were evaluated. The means show that for nearly all the attributes (except “relaxed-stressed”) the two pictures showing the ‘happy’ pig separately and in a straw pen are evaluated equally and more positively compared to the other pictures. This is followed by the ‘unhappy’ pig on straw. Further, the ‘unhappy’ pig separately is evaluated similarly to the ‘happy’ pig in the slatted floor setting. This holds true for all attributes apart from “brave–anxious” where the ‘unhappy’ pig seperately is evaluated as more anxious compared to the ‘happy’ pig on slatted floor. The ‘unhappy’ pig on slatted floor receives the most negative evaluations for all attributes.

**Table 4:** Mean comparison using ANOVA and post-hoc tests for the evaluation of the ʽhappyʼ and the ʽunhappyʼ pig presented separately or in the two pens (straw/slatted floor).

Word pair	Evaluations of the pigs						P-value from F-test
	ʽHappyʼ Pig (n = 528)	‘Happy’ pig on straw (N = 1019)	‘Happy’ pig on slatted floor (N = 1019)	‘Un-happy’ pig (n = 491)	‘Un-happy’ pig on straw (N = 1019)	‘Un-happy’ pig on slatted floor (N = 1019)	
^1^Satisfied–unsatisfied	2.57^a^ (1.05)	2.43^a^ (1.11)	3.45^b^ (1.12)	3.37^b^ (1.15)	3.00^c^ (1.20)	3.91^d^ (1.01)	<0.001
^1^Happy–unhappy	2.77^a^ (0.99)	2.63^a^ (1.06)	3.57^b^ (1.06)	3.54^b^ (1.09)	3.12^c^ (1.16)	4.00^d^ (0.97)	<0.001
^2^Relaxed–stressed	2.51^a^ (1.01)	2.37^b^ (1.00)	3.34^c^ (1.04)	3.17^c^ (1.04)	2.82^d^ (1.10)	3.60^e^ (0.98)	<0.001
^2^Active–inactive	2.78^a^ (1.06)	2.69^a^ (1.07)	3.43^b^ (1.03)	3.57^b^ (1.06)	3.12^c^ (1.09)	3.87^d^ (0.99)	<0.001
^1^Healthy–sick	2.24^a^ (0.93)	2.13^a^ (0.91)	2.77^b^ (0.97)	2.79^b^ (1.12)	2.51^c^ (1.06)	3.17^d^ (1.05)	<0.001
^1^Brave–anxious	2.71^a^ (0.95)	2.65^a^ (0.97)	3.23^b^ (0.98)	3.42^c^ (1.04)	3.13^b^ (1.02)	3.66^d^ (1.00)	<0.001

Evaluation on a five-point semantic differential scale ranging from 1 = most positive to 3 = neutral to 5 = most negative. Displayed are means and standard deviations (SD) in brackets. Comparison of means using ANOVA and post-hoc tests. Different letters indicate differences according to post-hoc tests.

^1^Variance heterogeneity is assumed

^2^Variance homogeneity is assumed.

Source: Own calculations.

The evaluations of the pens (Table 5) reveal that in terms of species-appropriateness and naturalness the three pictures showing the straw pen are evaluated equally and the three pictures showing the slatted floor pen are evaluated equally as well. The straw pen receives more positive values compared to the slatted floor pen on all pictures. Looking at how comfortable the pens are rated by participants, the most positive evaluation is given to the straw pen with the ‘happy’ pig, followed by the other two straw pens (separately and with the ‘unhappy’ pig). Again here, the slatted floor pen is evaluated always the same and more negatively. The straw pen with the ‘happy’ pig is evaluated as the most future-proof and the cleanest pen (same evaluation in terms of cleanliness for the straw pen with the ‘unhappy’ pig), followed by the straw pen separately and the straw pen with the ‘unhappy’ pig for future-proof. The slatted floor pictures are evaluated as being less future-proof (same for all three pictures) and more dirty compared to the straw pen settings.

**Table 5:** Mean comparison using ANOVA and post-hoc tests for the evaluation of the straw and slatted floor pen presented separatly or with a pig (‘happy’/’unhappy’ pig).

Word pair	Evaluations of the pens						P-value from F-test
	Straw pen (n = 521)	Straw pen with ‘happy’ pig (n = 1019)	Straw pen with ‘unhappy’ pig (n = 1019)	Slatted floor pen (n = 498)	Slatted floor pen with ‘happy’ pig (n = 1019)	Slatted floor pen with ‘unhappy’ pig (n = 1019)	
^1^Species-appropriate–not species-appropriate	2.55^a^ (1.20)	2.43^a^ (1.16)	2.53^a^ (1.18)	4.01^b^ (1.16)	4.09^b^ (1.11)	4.16^b^ (1.06)	<0.001
^2^Natural–unnatural	2.57^a^ (1.20)	2.44^a^ (1.17)	2.55^a^ (1.22)	4.02^b^ (1.15)	4.0b^b^ (1.10)	4.15^b^ (1.05)	<0.001
^2^Comfortable–uncomfortable	2.67^a^ (1.18)	2.39^b^ (1.10)	2.57^a^ (1.15)	3.97^c^ (1.13)	3.96^c^ (1.12)	4.09^c^ (1.04)	<0.001
^1^Future-proof– not future-proof	2.76^a^ (1.13)	2.53^b^ (1.15)	2.64^ab^ (1.16)	3.87^c^ (1.13)	3.85^c^ (1.14)	3.26^c^ (1.30)	<0.001
^2^Clean–dirty	2.57^a^ (1.18)	2.26^b^ (1.07)	2.29^b^ (1.08)	3.17^c^ (1.35)	2.90^d^ (1.28)	3.00^cd^ (1.27)	<0.001

Evaluation on a five-point semantic differential scale rangning from 1 = most positive to 3 = neutral to 5 = most negative. Means are displayed and standard deviations (SD) in brackets. Comparison of means using ANOVA and post-hoc tests. Different letters indicate differences according to post-hoc tests.

^1^Variance heterogeneity is assumed

^2^Variance homogeneity is assumed

Source: Own calculations.

### Effects influencing the evaluation of pig and pen in the combined pictures

Table 6 shows the main effects and interactions included in the model that analyzes the influences on the evaluation of the pig in the combined pictures. The pen has the largest influence on pig evaluation, followed by the pig itself, and by participants’ belief in pig mind and whether participants stated to not have any connection to agriculture. Whether the participants belong to split one or two (separate or combined pictures first) does not influence pig evaluation. The first combined picture that the respondents saw also does not infleunces pig perception. Furthermore, the interaction of Pig x Pen does not influence the evaluation of the pig.

**Table 6:** Type III tests and estimates of fixed effects in the linear mixed model on pig evaluation in the pictures.

Effect		Type III tests of fixed effects (F-values)	Estimates of fixed effects	SE	T-values
Intercept		7808.43***	3.60	0.09	41.31***
Pig		452.26***	-0.42	0.03	-13.92***
Pen		1295.14***	-0.73	0.03	-24.33***
Split		0.21	0.02	0.04	-0.45
First Picture		2.42			
	1 (unhappy/straw)		-0.01	0.06	-0.09
	2 (happy/straw)		0.13	0.06	2.31*
	3 (happy/slatted		0.03	0.06	0.51
	4 (unhappy/slatted)		0	-	-
Grew up on a farm		2.97	0.12	0.07	1.72
No connection to agriculture		4.71*	-0.10	0.04	-2.17*
Belief in Pig Mind I		9.29**	0.06	0.02	3.05**
Belief in Pig Mind II		25.75***	-0.10	0.02	-5.07***
Pig x Pen		2.52	-0.07	0.04	-1.59

Dependent variable: index of pig evaluation including evaluation of six word pairs (satisfied—unsatisfied, happy—unhappy, relaxed—stressed, active—passive, healthy—sick, brave—anxious)

Model covariance structure: scaled identity

*** = p ≤ 0.001

** = p ≤ 0.01

* = p ≤ 0.05

SE = standard error

Source: Own calculations

Table 7 shows the main effects and interaction included in the linear mixed model that analyzes the evaluation of the pen. The largest effect on pen evaluation is again the pen, followed by the pig and participants’ belief in pig mind. Contrasting the model of pig evaluation, the split, indicating whether participants saw the combined or single pictures first, shows an effect on pen evaluation in the combined pictures. Pariticpants’ connections to agriculture (whether they grew up on a farm or have no connection to agriculture) further influence pen evaluation. However, the first combined picture that participants saw does not effectpen evaluation. Also, the interaction between Pig and Pen does not influence pen evaluation.

**Table 7:** Type III tests of fixed effects and estimates of fixed effects in the linear mixed model on pen evaluation in the pictures.

Effect		Type III tests of fixed effects (F-values)	Estimates of fixed effects	SE	T-values
Intercept		6298.08***	3.66	0.10	37.78***
Pig		19.76***	-0.09	0.03	-2.94**
Pen		3625.69***	-1.35	0.03	-42.38***
Split		5.09*	0.10	0.05	2.26*
First Picture		2.32			
	1 (unhappy/straw)		0.09	0.06	1.38
	2 (happy/straw)		0.12	0.06	1.80
	3 (happy/slatted		-0.03	0.06	-0.48
	4 (unhappy slatted)		0	-	-
Grew up on a farm		7.40**	0.22	0.08	2.72**
No connection to agriculture		11.24**	-0.17	0.05	-3.35**
Belief in Pig Mind I		12.65***	0.08	0.02	3.56***
Belief in Pig mind II		31.84***	-0.13	0.02	-5.64***
Pig x Pen		0.08	-0.01	0.05	-0.29

Dependent variable: index of pen evaluation including evaluation of five word pairs (species-appropriate–not species-appropriate, natural–unnatural, comfortable–uncomfortable, future-proof–not future-proof, clean–dirty)

Model covariance structure: scaled identity

*** = p ≤ 0.001

** = p ≤ 0.01

* = p ≤ 0.05

SE = standard error

Source: Own calculations

Table 8 shows the effects of picture composition (the pig and the pen) on pig and pen evaluation resulting from the models presented above. It shows that respondents’ evaluations of both, the two pigs and two pens, differ from each other. The evaluation mean of the ‘happy’ pig is more positive compared to the ‘unhappy’ pig. The straw pen also improves picture evaluation compared to the slatted floor pen. In addition, the difference between the LS means is higher between the two pens than the two pigs.

**Table 8:** Effects of pig and pen on pig and pen evaluation (1 = positive evaluation, 5 = negative evaluation) in the combined pictures showing a pig (‘happy’ or ‘unhappy’) in a pen (straw or slatted floor) using LS Means comparison with F-test.

	Picture elements		SE	P-value
	Happy pig	Unhappy Pig		
Index of pig evaluation LS Means	2.88	3.33	0.01	<0.001
Index of pen evaluation LS Means	3.09	3.19	0.02	<0.001
	Straw pen	Slatted floor pen		
Index of pig evaluation LS Means	2.73	3.49	0.01	<0.001
Index of pen evaluation LS Means	2.47	3.82	0.01	<0.001

Source: Own calculations.

Table 9 shows the effects of participants’ connections to agriculture (either grew up on a farm or not, and stating to have no connection to agriculture, or not) and the effect of picture order (split) on pig and pen evaluation resulting from the two models. Growing up on farm improves both pig and pen evaluation in the models. Having no connection to agriculture leads to a more negative evaluation of pigs and pens in the models. The split only effects picture evaluation in case of pen evaluation with a more positive evaluation from participants in split 2, who saw the combined pictures first.

**Table 9:** Effects of growing up on a farm, connection to agriculture and picture order on pig and pen evaluation (1 = positive evaluation, 5 = negative evaluation) in the combined pictures showing a pig in a pen using LS Means comparison with F-test.

	Effects		SE	P-value
	Grew up on a farm	Did not grow up on a farm		
Index of pig evaluation LS Means	2.93	3.13	0.01	<0.001
Index of pen evaluation LS Means	2.83	3.18	0.01	<0.001
	No connection to agriculture at all	Somehow a connection to agriculture		
Index of pig evaluation LS Means	3.16	3.04	0.01	<0.001
Index of pen evaluation LS Means	3.23	3.03	0.01	<0.001
	Split 1	Split 2		
Index of pig evaluation LS Means	3.11	3.11	0.01	0.825
Index of pen evaluation LS Means	3.20	3.08	0.01	<0.001

Source: Own calculations.

## Discussion

Confirming the results of the pretest, the evaluation of the ʽhappyʼ and the ʽunhappy’ pig revealed more positive values for the perceived welfare of the ʽhappyʼ pig. Considering the body language of the pig, it is not surprising that a more upright position of the ‘happy’ pig’s head, with the ears standing up is likely to be more positively assessed than a downward facing pig with hanging ears. Indeed, researchers relate ear postures of farm animals to their emotional states (for example for sheep: [24]). Nevertheless, indicators for the reliable identification of emotional states in farm animals are not existent yet [24] and the real affective state of the pig at the point of picture-taking remains uncertain. Further, for the purpose of this study, we concentrated on the question in which way a more positive or negative perception of the pig’s facial expression influenced picture evaluation, rather than evaluation if people can correctly interpret pig emotions.

Regarding the evaluation of the two pens, it could be observed that the slatted floor is perceived more negatively than straw bedding. The negative perception of the slatted floor in this study is striking and is in line with the lack of acceptance for this husbandry system by the majority of German citizens [39], as well as those in other European countries [9]. In contrast, the more positive evaluation of the straw pen is in line with the finding that litter bedding has been shown to have a strong positive influence on the evaluation of husbandry systems by people from the broader public [10]. Nevertheless, the level of dirt on the wall differs between the two pictures with the slatted floor pen picture showing higher and darker amounts of dirt. We are therefore not able to clearly distinguish wether the more negative evaluation of the slatted floor setting is due to the floor type or to the level of dirt on the wall, which clearly limits the validity of our results.

We showed that for the depiction of pigs in either a slatted floor pen or a straw pen (that also differed in dirt level on the wall), the barn setting had a larger influence on picture evaluation compared to the pigs’ expressions. These findings are in line with studies which suggest that picture background influences the perception of the object [40], and further that the perception of animals is influenced by the context in which they are depicted [1, 2, 3, 41, 42]. When the general public assesses husbandry systems for pigs, housing and floor type significantly contribute to their evaluation [9, 10, 11] and this holds also true for the picture evaluations in our study. Accordingly, the welfare of both pigs is rated more negatively when the pig is standing on a slatted floor with a dirty wall rather than on straw bedding with a cleaner wall. Even the ʽunhappyʼ pig on straw is evaluated better than the ʽhappyʼ pig on slatted floor. Here, a negatively perceived husbandry system such as the slatted floor pen [5, 39] (or the higher amount of dirt on the wall) causes a negative evaluation of the pig’s welfare, regardless of the animalsʼ facial expression. Thus, the observer’s experience and knowledge about the world clearly affect their perception of pictures [15, 43, 44]. This is also supported by the fact that respondents belief in pigs’ mind effects picture evaluation in our study. The level of participants’ connection to agriculture also influences picture evaluation with a closer relation to agriculture leading to a more positive perception of both the pigs and the pens, which is in line with other studies analyzing peoples’ attitudes towards agriculture [29; 30]. The influence of the split shows a more positive evaluation of the pen (but not of the pig) for split 2 participants who saw the combined pictures first. This might due to the possibility that people in split 2 do not have a reference picture in mind because they haven’t seen the single pictures at that point. Contrastingly, split 1 participants might use the single pictures they have seen as frame of reference for the combined pictures, leading to a more critical evaluation of the pen. Why this effect only occurs for the evaluation of the pen and not for the pig remains unanswered.

Our results underline that both, picture composition (bottom-up) factors as well as top-down factors, such as as the viewers attitude towards pigs, influence the perception of pictures [45]. The positive effect of straw and the more positive evaluation of the animal’s welfare in the straw setting might not only be explained by more positive images of straw systems regarding the animal’s wellbeing [7, 9, 10], but also by a generally more positive association of pigs with straw. People visiting pig farms experience nostalgia when smelling straw, which possibly leads to more positive reactions [7]. Straw is further considered to be important for housing systems for pigs by the general public because of its naturalness, possibilities for playing and its bedding properties [7]. Due to the different levels of dirt in the pictures used in the study, the better evaluation of the straw pen might also trace back to the slightly cleaner pen compared to the slatted floor pen.

The effect that different conclusions are drawn about the well-being of the pigs based on either the straw or the slatted floor setting, despite non-changing facial expression, is similarly known from filmmakers’ editing techniques. Different contexts (such as neutral versus emotional contextual movies) can alter the perception of facial expressions and mental states of the shown actors, a fact which is known as the Kuleshov effect ([46]; see also [47]). Mobbs et al. [47] were able to show through an experiment that identical facial expressions are perceived differently when paired with either neutral or emotional contextual movies. Similarly, the design of the pen influences the evaluation of the pig that lives in it depending on already existing or just formed opinions about that system. Finally, that the environment is strongly linked to the well-being of the animal being exposed to that environment seems likely and could explain the strong influence of the pen on picture evaluation in our study.

The pig and its expression also influenced pig and pen evaluation, but with minor strength. As the pig was probably in the focus of the picture inspection [48, 49, 50], conclusions related to the animal’s well-being were drawn from the pig’s body language and facial expression. However, the effect of the pig on pig and pen evaluation is lower than the effect of the pen tested in our study. Behavioral studies suggest that the context in which faces are shown has the largest effect when the clarity of facial expression is low, while the clarity of the context is high [47, 51, 52]. This might explain the strong effect of the pen, as well as the comparably smaller effect of the pig. Even though respondents obviously drew conclusions from the pig’s body language and facial expression, it might have been easier to evaluate the pen than the affective state of the pig presented in the picture, especially when opinions on husbandry systems might be present. Additionally, it should be considered that the expression of the pig is only a snapshot and could thus change within a matter of seconds, whereas the environment is consistent and enduring. Respondents could possibly recognize this point and therefore draw conclusions rather from the steadier element in the picture, which is the pen. It can therefore be summarized that the expression of the pig contributes to picture evaluation and the perception of the pig’s welfare, but cannot dramatically change the evaluation of the pen.

## Conclusions

Regarding the perception of farmed pigs in pictures, the context in which an animal is presented seems to influence the public perception of the scene. Husbandry systems for farm animals play a dominant role in the context of perceived animal welfare in modern communication processes pertaining to agriculture. Thereby, positive or negative (pre-existing) attitudes towards husbandry systems might alter the overall evaluation of animal welfare. Contrastingly, the pig’s influence on the evaluation of the barn is comparably small, suggesting that a more positively perceived animal does not have the power to overcome negative expectations towards contentious husbandry systems, or that more negatively perceived animals cannot overcome positive expectations, at least in our study design. This needs to be taken into consideration for the discussion surrounding farm animal welfare and the perception of pictures showing animals in their environment in the media.

### Limitation

As a limitation in study design it must be noted that only one picture for each husbandry system (one with straw and one with slatted floor) has been tested. In order to draw broader conclusions about the systems in general, more different pictures showing both systems should have been used. This holds also true for the two pigs. Further, the two pens show different degrees of wall contamination which could also possibly have influenced picture evaluation. In future studies, the level of dirt on the walls, as well as all other potentially confounding features, should be either eliminated or kept exactly the same between the pictures in order to be able to clearly identify the effects of pen type on picture evaluation. Further, the two splits that participants were randomly assigned to differed in peoples’ connection to agriculture. In future studies it should be controlled for this aspect if samples are split.

## Supporting information

S1 File Output factor pig and pen. (XLSX)

S2 File Output factor belief in pig mind. (XLSX)

S3 File Output model. (XLSX)

## References

[1] Maple TL. Environmental psychology and great ape reproduction. International Journal for the Study of Animal Problems. 1983; 4: 295–299.

[2] Rhoads DL, Goldsworthy RJ. The effects of zoo environments on public attitudes toward endangered wildlife. International Journal of Environmental Studies. 1979; 13(4): 283–287.

[3] Finlay T, James LR, Maple TL. People’s perceptions of animals. The influence of zoo environment. Environment and Behavior. 1988; 20(4): 508–528.

[4] Wemelsfelder F, Nevison I, Lawrence AB. The effect of environmental background on qualitative assessments of pig behavior. Animal Behaviour. 2009; 78(2): 477–484.

[5] Lassen J, Sandøe P, Forkman B. Happy pigs are dirty!–conflicting perspectives on animal welfare. Livestock Science. 2006; 103(3): 221–230.

[6] Hall C, Sandilands V. Public attitudes to the welfare of broiler chickens. Animal Welfare 2007; 16(4): 499–512.

[7] Boogaard BK, Boekhorst LJS, Oosting SJ, Sørensen JT. Socio-cultural sustainability of pig production: Citizen perceptions in the Netherlands and Denmark. Livestock Science. 2011a; 140(1): 189–200.

[8] Ryan EB, Fraser D, Weary DM. Public attitudes to housing systems for pregnant pigs. PloS ONE 2015; 10(11): e0141878 10.1371/journal.pone.0141878 26559417 PMC4641725

[9] Krystallis A, de Barcellos MD, Kügler JO, Verbeke W, Grunert KG. Attitudes of European citizens towards pig production systems. Livestock Science. 2009; 126(1): 46–56.

[10] Verbeke W, Pérez-Cueto JA, de Barcellos MD, Krystallis A, Grunert KG. European citizen and consumer attitudes and preferences regarding beef and pork. Meat Science. 2010; 84(2): 284–292. 10.1016/j.meatsci.2009.05.001 20374787

[11] Janssen M, Rödiger M, Hamm U. Labels for Animal Husbandry Systems Meet Consumer Preferences: Results from a Meta-analysis of Consumer Studies. Journal of Agricultural and Environmental Ethics. 2016; 29(6) 1071–1100.

[12] Sørensen BT, de Barcellos MD, Olsen NV, Verbeke W, Scholderer J. Systems of attitudes towards production in the pork industry. A cross-national study. Appetite. 2012; 59(3): 885–897. 10.1016/j.appet.2012.08.021 22940421

[13] Eurostat. Statistics Explained. File: Statistics on the pig population, slaughtering and pigmeat production, 2013. Database: Eurostat [Internet]. Accessed: http://ec.europa.eu/eurostat/statistics-explained/index.php/File:Statistics_on_the_pig_population,_slaughtering_and_pigmeat_production,_2013.png, (30.09.2016).

[14] Statistisches Bundesamt. Landwirtschaftszählung 2010. Statistisches Bundesamt (Destatis), Wiesbaden, 2010. Database: Destatis [Internet]. Accessed: https://www.destatis.de/DE/ZahlenFakten/Wirtschaftsbereiche/LandForstwirtschaftFischerei/Landwirtschaftszaehlung2010/Tabellen/9_2_LandwBetriebHaltungsplaetzeSchweine.html, 2010, (30.09.16).

[15] Davenport JL, Potter MC. Scene consistency in object and background perception. Psychological Science. 2004; 15(8): 559–564. 10.1111/j.0956-7976.2004.00719.x 15271002

[16] Schirmer A, Seow CS, Penney TB. Humans process dog and human facial affect in similar ways. PloS ONE 2013; 8(9): e74591 10.1371/journal.pone.0074591 24023954 PMC3762775

[17] Kujala MV, Somppi S, Jokela M, Vainio O, Parkkonen L. Human Empathy, Personality and Experience Affect the Emotion Ratings of Dog and Human Facial Expressions. PloS ONE 2017; 12(1): e0170730 10.1371/journal.pone.0170730 28114335 PMC5257001

[18] Konok V, Nagy K, Miklósi Á. How do humans represent the emotions of dogs? The resemblance between the human representation of the canine and the human affective space. Applied Animal Behaviour Science. 2014; 162(2015): 37–46.

[19] Dalla Costa M, Minero M, Lebelt D, Stucke D, Canali E, Leach MC. Development of the horse grimace scale (HGS) as a pain assessment tool in horses undergoing routine castration. PLoS ONE. 2014; 9(3): e92281 10.1371/journal.pone.0092281 24647606 PMC3960217

[20] Langford DJ, Bailey AL, Chanda ML, Clarke SE, Drummond TE, Echols S. et al Coding of facial expressions of pain in the laboratory mouse. Nature Methods. 2010; 7(6): 447–449. 10.1038/nmeth.1455 20453868

[21] Sotocinal SG, Sorge RE, Zaloum A, Tuttle AH, Martin LJ, Wieskopf J.S., et al The Rat Grimace Scale: A partially automated method for quantifying pain in the laboratory rat via facial expressions. Molecular Pain. 2011; 7(55): 1–10. 21801409 10.1186/1744-8069-7-55 PMC3163602

[22] Rousing T, Wemelsfelder F. Qualitative assessment of social behavior of dariy cows housed in loose housing systems. Applied Animal Behaviour Science. 2006; 101(1): 40–53.

[23] Wemelsfelder F, Hunter AE, Paul ES, Lawrence AB. Assessing pig body language: Agreement and consistency between pig farmers, veterinarians, and animal activists. Journal of Animal Science. 2012; 90(10): 3652–3665. 10.2527/jas.2011-4691 22745187

[24] Boissy A, Aubert A, Désiré L, Greiveldinger L, Delval E, Veissier I. Cognitive sciences to relate ear postures to emotions in sheep. Animal Welfare. 2011; 20(1): 47–56.

[25] Busch G, Weary DM, Spiller A, von Keyserlingk MAG. American and German attitudes towards cow-calf separation on dairy farms. PLoS ONE. 2017; 12(3): e0174013 10.1371/journal.pone.0174013 28301604 PMC5354428

[26] Knight S, Barnett L. Justifying attitudes towards animal use: a qualitative study of people’s views and beliefs. Anthrozoos. 2008; 21: 31–42.

[27] McFarland SG. Effects of order on survey responses. The Public Opinion Quarterly. 1981; 45(2): 208–215.

[28] Tourangeau R, Rasinski KA. Cognitive Processes underlying context effects in attitude measurement. 1988; 103(3): 299–314.

[29] Boogaard BK, Oosting SJ, Bock BB. Elements of societal perception of farm animal welfare: A quantitative study in The Netherlands. Livestock Science. 2006; 104(1–2): 13–22.

[30] Vanhonacker F, Verbeke W, Van Poucke E, Tuyttens FAM. Do citizens and farmers interpret the concept of farm animal welfare differently? Livestock Scuence. 2008; 116(1–3): 126–136.

[31] Hills AM. Empathy and belief in the mental experience of animals. Anthrozoos. 1995; 8: 132–142.

[32] Myers OE, Saunders CD, Birjulin AA. Emotional dimensions of watching zoo animals: An experience sampling study building on insights from psychology. Curator: The Museum Journal. 2004; 47(3): 299–321.

[33] Frewer LJ, Kole S, van de Kroon SMA, de Lauwere C. Consumer Attitudes towards the Development of Animal-Friendly Husbandry Systems. Journal of Agricultural and Environmental Ethics. 2005; 18(4): 345–367.

[34] Boogaard BK, Bock BB, Oosting SJ, Wiskerke JSC. The sociocultural sustainability of livestock farming: an inquiry into social perceptions of dairy farming. Animal. 2011b; 5(09): 1458–1466. 22440292 10.1017/S1751731111000371

[35] Clark B, Stewart GB, Panzone LA, Kyriazakis I, Frewer LJ. A Systematic Review of Public Attitudes, Perceptions and Behaviours Towards Production Diseases Associated with Farm Animal Welfare. Journal of Agricultural and Environmental Ethics. 2016; 29(3): 455–478.

[36] Zhang C, Conrad FH. Speeding in Web Surveys: The tendency to answer very fast and its association with straightlining. Survey Research Methods. 2014; 8(2): 127–135.

[37] Statista. Altersstruktur der Bevölkerung in Deutschland zum 31. Dezember 2015, 2015. Database: Statista [Internet]. Accessed: http://de.statista.com/statistik/daten/studie/1351/umfrage/altersstruktur-der-bevoelkerung-deutschlands/, (20.02.17).

[38] Statistisches Bundesamt. Bevölkerung auf Grundlage des Zensus 2011. Statistisches Bundesamt (Destatis), Wiesbaden, 2011. Database: Destatis [Internet]. Accessed: https://www.destatis.de/DE/ZahlenFakten/GesellschaftStaat/Bevoelkerung/Bevoelkerungsstand/Tabellen/Zensus_Geschlecht_Staatsangehoerigkeit.html, (20.02.17).

[39] Roosen J, Dahlhausen JL, Petershammer S. Acceptance of Animal Husbandry Practices: The Consumer Perspective. Proceedings in System Dynamics and Innovation in Food Networks. 2016; 260–267. Accessed: http://centmapress.ilb.uni-bonn.de/ojs/index.php/proceedings/article/view/1630/576, (24.10.16).

[40] Davenport JL. Consistency effects between objects in scenes. Memory and Cognition. 2007; 35(3): 393–401. 17691140 10.3758/bf03193280

[41] Sommer R. What do we learn at the zoo? Natural History. 1972; 81(7): 26–27, 84–85.

[42] Bitgood S, Benefield A, Patterson D, Lewis D, Landers A. Zoo visitors: can we make them behave. Annual Proceedings of the American Association of Zoological Parks and Aquariums. 1985; 419–432.

[43] Palmer SE. The effects of contextual scenes on the identification of objects. Memory and Cognition 1975; 3(5): 519–526. 10.3758/BF03197524 24203874

[44] Bar M. Visual objects in context. Nature Reviews Neuroscience. 2004; 5(8): 617–629. 10.1038/nrn1476 15263892

[45] Wedel M, Pieters R. A review of eye-tracking research in Marketing In: Malhotra NK, editor. Review of Marketing Research, Vol. 4 Bingley: Emerald Group Publishing Limited; 2008: 123–147.

[46] Kuleshov LV. Kuleshov on film: writings 1st ed Chicago, IL: University of California Press; 1974.

[47] Mobbs D, Weiskopf N, Lau HC, Featherstone E, Dolan RJ, Frith CD. The Kuleshov Effect: the influence of contextual framing on emotional attributions. Social Cognitive and Affective Neuroscience. 2006; 1(2), 95–106. 10.1093/scan/nsl014 17339967 PMC1810228

[48] Buswell GT. How people look at pictures: a study of the psychology of perception in art. Chicago, IL: University of Chicago Press; 1935.

[49] Yarbus AL. Eye movements and vision Ney York: Plenum Press; 1967.

[50] Kano F, Tomonaga M. How chimpanzees look at pictures: a comparative eye-tracking study. Proceedings of the Royal Society B: Biological Sciences. 2009; 276: 1949–1955. 10.1098/rspb.2008.1811 19324790 PMC2677242

[51] Ekman P, Friesen W, Ellsworth P. What are the relative contributions of facial behavior and contextual information to the judgment of emotion? In: Ekman P, editor. Emotion in the human face. New York: Cambridge University Press; 1982.

[52] Trope Y. Identification and Inferential Processes in Dispositional Attribution. Psychological Review. 1986; 93(3): 239–57.

